# Human–Computer Agreement of Electrocardiogram Interpretation for Patients Referred to and Declined for Primary Percutaneous Coronary Intervention: Retrospective Data Analysis Study

**DOI:** 10.2196/24188

**Published:** 2021-03-02

**Authors:** Aleeha Iftikhar, Raymond Bond, Victoria Mcgilligan, Stephen J Leslie, Charles Knoery, James Shand, Adesh Ramsewak, Divyesh Sharma, Anne McShane, Khaled Rjoob, Aaron Peace

**Affiliations:** 1 Computing Engineering and Build Environment Ulster University Belfast United Kingdom; 2 Centre for Personalised Medicine Ulster University Londonderry United Kingdom; 3 Cardiac Unit Raigmore Hospital Inverness United Kingdom; 4 Department of Cardiology Altnagelvin Hospital Western Health and Social Care Trust Londonderry United Kingdom; 5 Letterkenny University Hospital Letterkenny Ireland

**Keywords:** ECG interpretation, agreement between human and computer, primary percutaneous coronary intervention service, acute myocardial infarction, scan, electrocardiogram, heart, intervention, infarction, human-computer, diagnostic

## Abstract

**Background:**

When a patient is suspected of having an acute myocardial infarction, they are accepted or declined for primary percutaneous coronary intervention partly based on clinical assessment of their 12-lead electrocardiogram (ECG) and ST-elevation myocardial infarction criteria.

**Objective:**

We retrospectively determined the agreement rate between human (specialists called activator nurses) and computer interpretations of ECGs of patients who were declined for primary percutaneous coronary intervention.

**Methods:**

Various features of patients who were referred for primary percutaneous coronary intervention were analyzed. Both the human and computer ECG interpretations were simplified to either “suggesting” or “not suggesting” acute myocardial infarction to avoid analysis of complex heterogeneous and synonymous diagnostic terms. Analyses, to measure agreement, and logistic regression, to determine if these ECG interpretations (and other variables such as patient age, chest pain) could predict patient mortality, were carried out.

**Results:**

Of a total of 1464 patients referred to and declined for primary percutaneous coronary intervention, 722 (49.3%) computer diagnoses suggested acute myocardial infarction, whereas 634 (43.3%) of the human interpretations suggested acute myocardial infarction (*P*<.001). The human and computer agreed that there was a possible acute myocardial infarction for 342 out of 1464 (23.3%) patients. However, there was a higher rate of human–computer agreement for patients not having acute myocardial infarctions (450/1464, 30.7%). The overall agreement rate was 54.1% (792/1464). Cohen κ showed poor agreement (κ=0.08, *P*=.001). Only the age (odds ratio [OR] 1.07, 95% CI 1.05-1.09) and chest pain (OR 0.59, 95% CI 0.39-0.89) independent variables were statistically significant (*P*=.008) in predicting mortality after 30 days and 1 year. The odds for mortality within 1 year of referral were lower in patients with chest pain compared to those patients without chest pain. A referral being out of hours was a trending variable (OR 1.41, 95% CI 0.95-2.11, *P*=.09) for predicting the odds of 1-year mortality.

**Conclusions:**

Mortality in patients who were declined for primary percutaneous coronary intervention was higher than the reported mortality for ST-elevation myocardial infarction patients at 1 year. Agreement between computerized and human ECG interpretation is poor, perhaps leading to a high rate of inappropriate referrals. Work is needed to improve computer and human decision making when reading ECGs to ensure that patients are referred to the correct treatment facility for time-critical therapy.

## Introduction

### Background

According to the British Heart Foundation, circulatory diseases cause more than one-quarter (27%) of all deaths in the United Kingdom [1]. In the United Kingdom, more than 100,000 hospital admissions each year are due to heart attacks (280 admissions per day) [1]. Acute coronary syndrome occurs due to a restriction in blood flow in the coronary arteries [2]. Acute coronary syndromes are subdivided into (1) ST-elevation myocardial infarctions, (2) non–ST-elevation myocardial infarctions, and (3) unstable angina [3]. ST-elevation myocardial infarction is generally more serious when there is total occlusion of a coronary blood vessel leading to extensive damage to a large area of the heart [4]. Once a blocked artery is suspected, a patient is typically referred for reperfusion therapy which can include a primary percutaneous coronary intervention [5]. The preferred treatment for an acute myocardial infarction with ST-segment elevation is angioplasty (primary percutaneous coronary intervention) given that this is an effective therapy for opening occluded arteries [6-8]. The admission criteria for primary percutaneous coronary intervention are often variable and partly based on electrocardiogram (ECG) interpretation and patient symptoms, hence not all referrals are accepted. Even if ST-elevation myocardial infarction is present, ECG interpretation can be difficult because of different factors, including misleading computerized interpretations, signal noise, poor confidence or competency in reading ECGs, human error, and indeed, borderline ECGs (not precisely normal, but not significantly abnormal either), that make it difficult for clinicians to make a binary decision. A strict criterion may result in patients with acutely occluded coronary arteries not getting the treatment in time. It has been reported that several patients not meeting ST-elevation myocardial infarction criteria who were nevertheless referred for primary percutaneous coronary intervention did indeed require angioplasty [9].

ECG interpretation is central to deciding whether patients should be declined or accepted for primary percutaneous coronary intervention. The ECG is the most widely used diagnostic tool for patients with suspected acute myocardial infarction [10,11]. Many prehospital protocols require the acquisition of a single 12-lead ECG when assessing a patient for a ST-elevation myocardial infarction or ischemia. However, if necessary, a second or third prehospital ECG is recorded to correctly identify a ST-elevation myocardial infarction due to the number of ECGs (15% in [5]) that are nonspecific, ambiguous, and perhaps borderline [5]. When arriving at an emergency, paramedics are often first to record and interpret the ECG. Different studies [12,13] have been conducted to compare ECG interpretation accuracy between paramedics and physicians. Mencl et al [12] found no correlation between training, experience, or confidence in the ability of paramedics to recognize ST-elevation myocardial infarctions. The paramedics in the study were only able to identify inferior ST-elevation myocardial infarctions and normal ECGs; paramedics' ECG interpretations cannot be solely relied on (low sensitivity and specificity) for activating the catheterization laboratory (CathLab), in which diagnostic imaging equipment used to visualize the arteries and the chambers of the heart and to treat any stenosis or abnormality, in a primary percutaneous coronary intervention service [12].

Identification of patients with acute myocardial infarction continues to be challenging, especially when automated ECG interpretation is inconclusive or misleading. However, a study [13] has shown that, when the ECG exhibits vagueness, clinician input (using the internet) can improve diagnostic performance and reduce time to treatment. It is well documented that misinterpretation of the ECG can lead to incorrect decision making regarding treatment, such as false activations (rates of up to 36% [14]) or patients being declined. According to Degheim et al [15], 12.5% of all CathLab activations were false activations for misinterpreted ST-elevation myocardial infarction. These false activations have both clinical and financial costs.

### Prior Work

Given the challenges of reading ECGs, computer interpretation has been used for many years to assist human interpreters. In a retrospective cross-sectional study [16] of 200 prehospital ECGs, computer interpretation for detecting ST-elevation myocardial infarction achieved a specificity of 100% (100/100; 95% CI 0.96-1.00) and a sensitivity of 58% (58/100; 95% CI 0.48-0.67). This illustrates that this computer algorithm would have incorrectly declined 42% of patients but had zero inappropriate activations [16]; the most common incorrect computer statements for false negatives were “data quality prohibits interpretation” and “abnormal ECG unconfirmed.” Another study [17] concluded that computer-interpretation failed to identify a number of patients with ST-elevation myocardial infarction. This shows that prehospital computerized ECG interpretation is suboptimal for ST-elevation myocardial infarction detection and should not be used as a single method for prehospital activation of the CathLab. Cardiologists are the most accurate diagnosticians and are the least likely to falsely activate the CathLab [18]. Nevertheless, other physicians, paramedics, and specialized nurses (activator nurse) are expected to competently read ECGs.

### Study Goals

Having summarized the research to date, we have identified that ECG interpretation is challenging for both humans and computers, and there is a need to better understand the characteristics of the patients who are declined for primary percutaneous coronary intervention, especially given that there are a number of likely false negatives (patients who are declined but needed an emergency intervention).

We aimed to analyze agreement between computer and human (activator nurses) ECG interpretations for patients who were referred to but declined for primary percutaneous coronary intervention.

## Methods

### Data Set

This study involved an analysis of an anonymized data set from Altnagelvin Hospital (Northern Ireland, United Kingdom) of consecutive patients who were declined for primary percutaneous coronary intervention from January 2015 to December 2017. The total study population consisted of 1464 patients who were referred but declined for a primary percutaneous coronary intervention.

### Data Collection

When paramedics suspect acute myocardial infarction based on ECG findings, they contact the primary percutaneous coronary intervention department at the hospital and describe the symptoms and ECG findings to an activator nurse. The activator nurse routinely records this referral using a paper-based form, which is then digitized to a spreadsheet. Therefore, the data contained some inconsistencies and missing values.

### Data Analysis

All statistical analyses were performed using R (version 3.5.2, RStudio). The time-series visualization of interpretations was generated using an R package for visual analytics (ggplot2; version 3.3.2). Data were interrogated for missing values and completeness. There were no missing values in the most important data columns (ie, computer ECG interpretation, activator nurse ECG interpretation); however, to overcome data inconsistencies, the required fields were manually cleaned. There were typographical issues such as the inconsistent use of mixed upper and lower case, spelling mistakes, use of shorthand, and abbreviations used in the computer and human ECG interpretation columns. Comparisons between the distinct groups were investigated for significance using chi-square tests for categorical dichotomous variables. One-tailed Student *t* or Mann-Whitney tests were used for continuous variables depending upon whether the variables were normally distributed. Logistic multivariate regression analysis was performed on independent variables such as gender, age, out of hours, chest pain, activator nurse interpretation, computer interpretation, and computer and activator nurse agreement where the response variables included 30-day and 1-year mortality (encoded as 1 or 0, where 1=mortality). We also investigated mutual agreement and disagreement over the 24-hour day. To analyze the agreement between the computer and activator nurse, all interpretations were simplified and re-encoded as either suggesting or not suggesting acute myocardial infarction. To achieve this binary encoding of ECG interpretations, 3 medical doctors (AP, SL, and CK—2 of whom were clinical lead and consultant cardiologists) reviewed the original interpretations. The 3 medical doctors independently reclassified these statements as either suggesting acute myocardial infarction or not suggesting acute myocardial infarction, then they met as a team to arrive at consensus when there were discrepancies.

### Ethical Aspects

Permission for the study was obtained from the Regional Ethical Review Board (IRAS 251710) of the National Health Service Office for Research Ethics Committees Northern Ireland. The study complied with the Declaration of International Research Integrity Association. After the study received ethical approval for secondary data analysis, the staff nurse removed all personal identifiable information such as names, date of birth, and unique patient identifiers.

## Results

### Activator Nurse and Computer ECG Interpretations

**Figure 1:**
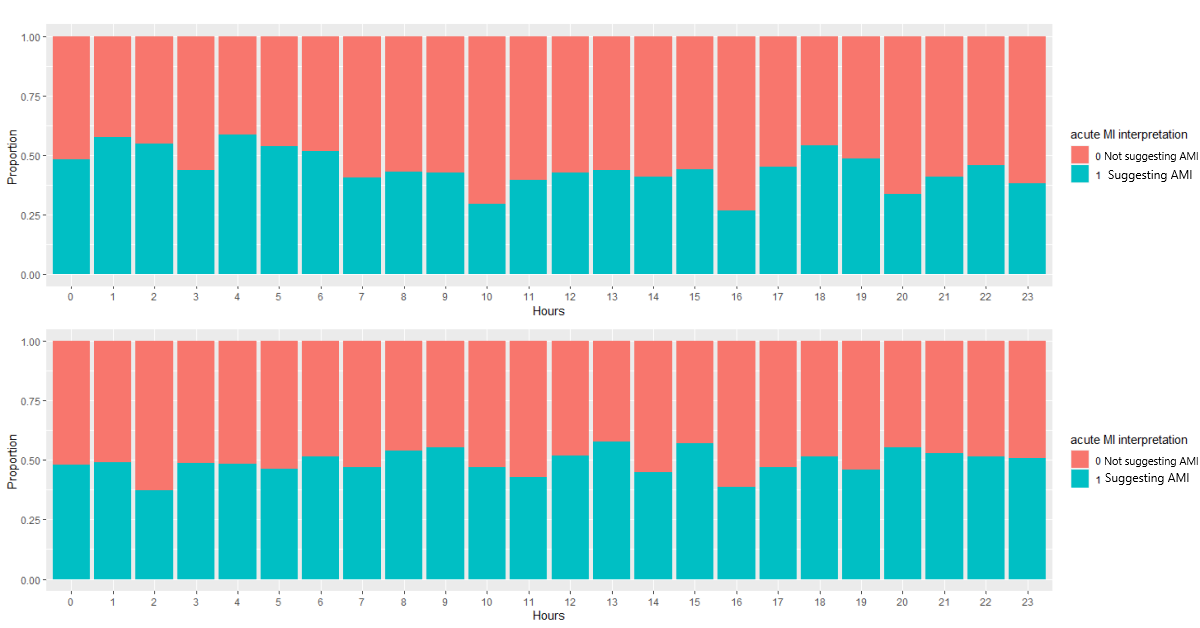
(a) Activator nurse and (b) computer interpretations of acute myocardial infarction rate by the hour. AMI: acute myocardial infarction; MI: myocardial infarction.

The computer suggested acute myocardial infarction more often than the activator nurses (722/1464, 49.3% vs 634/1464, 43.3%; *P*=.001). Figure 1 depicts the acute myocardial infarction interpretation rate per hour for both the activator nurses and the computer. The highest relative rate of acute myocardial infarction interpretation by activator nurses occurred at 1 AM (26/45, 57.8%) and 4 AM (17/29, 58.6%). The activator nurses seemed to interpret more acute myocardial infarctions during the middle of the night (12 AM to 6 AM) with a mean of 53% (SD 5.3%) compared to during daytime hours (mean 41%, SD 6.6%; *P*=.001). In contrast, computer interpretation did not show much variation with respect to hours of the day; for the middle of the night (12 AM to 6 AM), the average acute myocardial infarction interpretation rate was a mean of 47% (SD 4.7%) compared with a mean 50% (SD 5.2%) for the daytime hours. There was slightly more variation in activator nurse interpretations than in those of the computer over the hours of the day.

### Activator Nurse and Computer Overall Agreement

**Figure 2:**
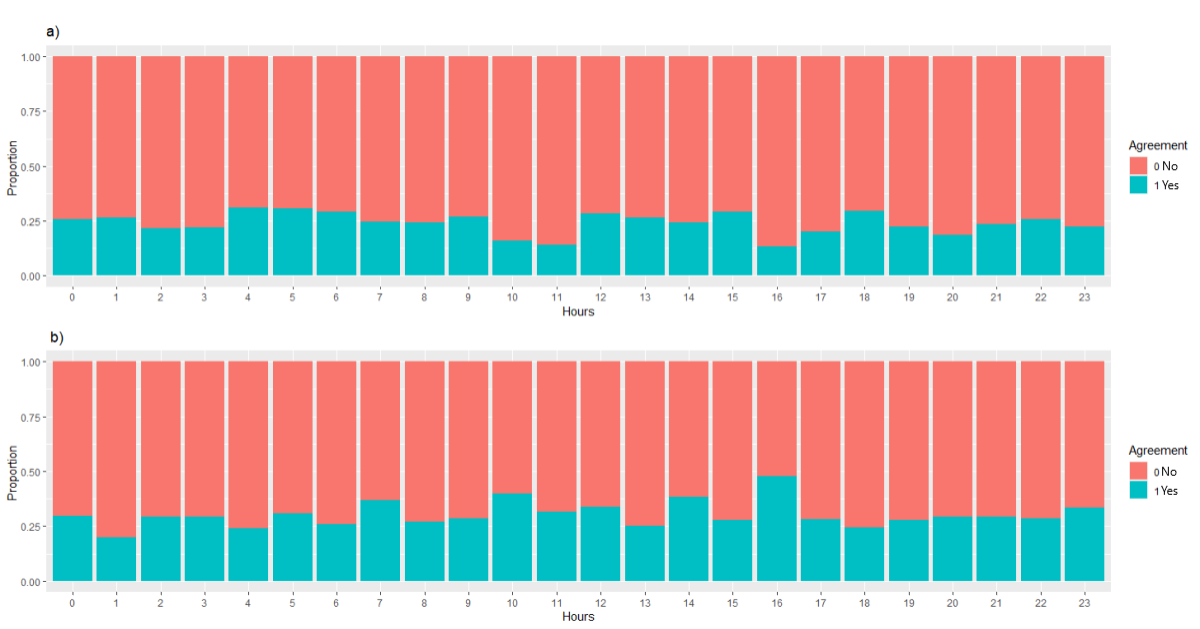
Activator nurse and computer agreement of (a) acute myocardial infarction and (b) not acute myocardial infarction. AMI: acute myocardial infarction; MI: myocardial infarction.

**Figure 3:**
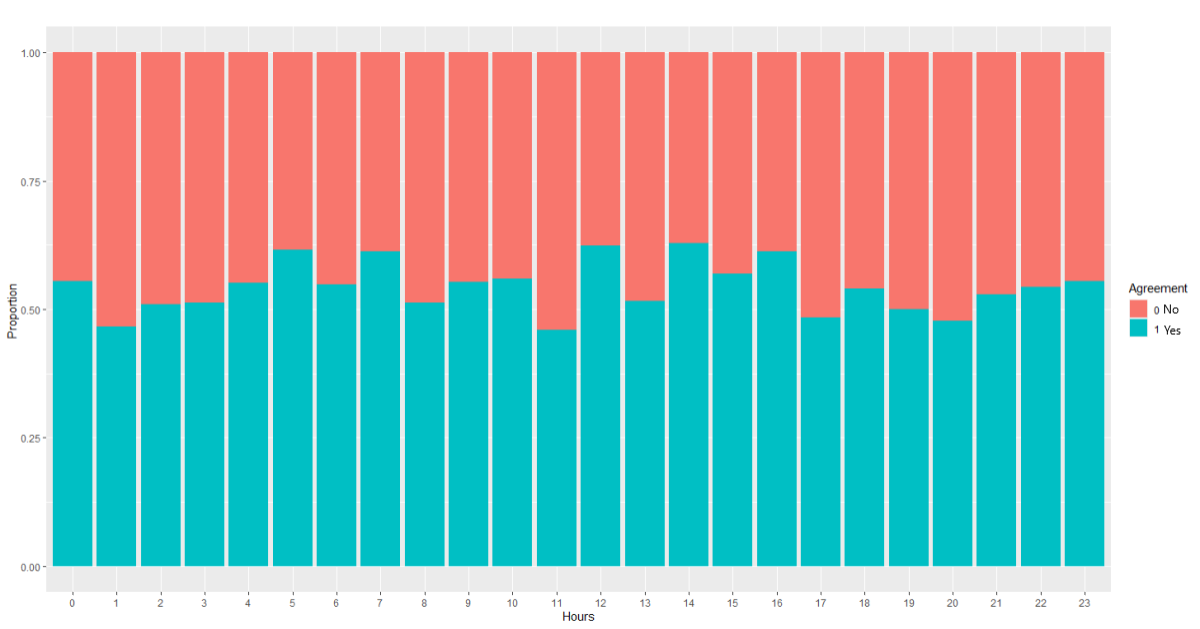
Activator nurse and computer agreement by the hour.

The human and computer ECG interpretations agreed for 54.1% of patients (792/1464; *P*<.001). This statistic includes suggesting and not suggesting acute myocardial infarction (Figure 2). The human–computer agreement rates were analyzed per hour; Figure 3 shows that the maximum agreement occurred at 12 PM and 2 PM during the daytime. Whereas in the middle of the night, the peak agreement occurred at 5 AM and 7 AM. Figure 2b shows that there was more variation in activator nurse and computer agreement not suggesting acute myocardial infarction than in those suggesting acute myocardial infarction (mean 57%, SD 7.5% vs mean 43% SD 4.7%; *P*<.001). There was more uncertainty *out of hours* when compared to *in hours*. Activator nurses suggested more acute myocardial infarctions during the middle of the night than in the daytime.

### Activator Nurse and Computer Overall Disagreement

The analysis of disagreement between human and computer interpretations was performed by first analyzing instances where activator nurses suggested acute myocardial infarction and the computer did not, and then vice versa. Maximum disagreement occurred at 11 AM.

### Activator Nurse Suggested Acute Myocardial Infarction

**Figure 4:**
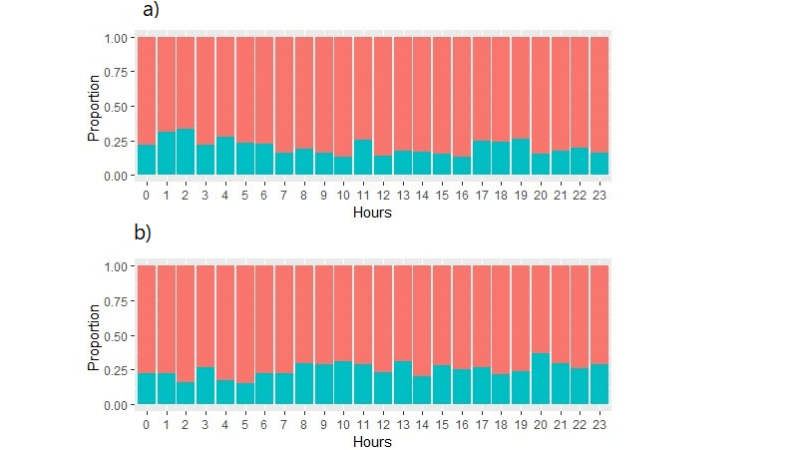
(a) Activator nurse interpretation suggesting acute myocardial infarction and computer disagreed; (b) computer interpretation suggesting acute myocardial infarction and activator nurse disagreed.

The number of patients for whom the activator nurse suggested acute myocardial infarction but the computer did not were selected and displayed per hour. The total number of such instances was 292/1464 (19.9%). Activator nurse interpretations suggested acute myocardial infarctions and the computer interpretation disagreed for more patients during the middle of the night (between 1 AM and 2 AM; Figure 4a).

### Computer Suggested Acute Myocardial Infarction

Computer interpretation suggested acute myocardial infarction and the corresponding activator nurses’ interpretation disagreed for 26.0% of patients (380/1464). The maximum disagreement occurred in the evening at 8 PM (*P*<.001; Figure 4b).

### Analysis of Other Variables

#### Patients With Chest Pain

**Figure 5:**
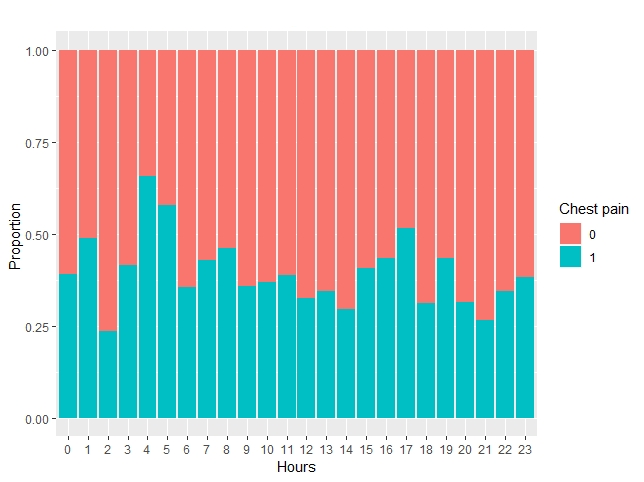
Proportion of patients with chest pain by the hour.

More males (1002/1464, 68.4%) were referred to primary percutaneous coronary intervention than females. More than half (769/1464, 52.5%) of the patients had either chest pain (n=556) or resolved chest pain (n=213). Most of these patients were male (385/556, 69.2%). More patients reported chest pain during the middle of the night (4 AM to 5 AM: 34/55, 61.8%; *P*=.02; Figure 5).

Logistic regression analysis was performed on independent variables including gender, age, out of hours, chest pain, activator nurse interpretation, computer interpretation, and computer–activator nurse agreement with the response variables being 30-day (Table 1) and 1-year mortality (Table 2). Age and chest pain were the only independent variables that were statistically significant (*P*<.001) for predicting mortality after 30 days or 1 year. Another trending variable was out of hours which increased the chance of mortality within 1 year (odds ratio [OR] 1.41, 95% CI 0.95-2.11). Being referred out of hours was more predictive for 1-year mortality than 30-day mortality. Being older (OR 1.07, 95% CI 1.05-1.09) increased the probability of 30-day and 1-year mortality. Activator nurse and computer agreement of acute myocardial infarction and having chest pain reduced the odds of mortality after 1 year. The odds of mortality within 30 days and 1 year of referral were lower in patients with chest pain compared to those patients without chest pain.

**Table 1:** Odds ratios of variables derived from multiple logistic regression where the response variable was mortality after 1 year.

Variable	Odds ratio (95% CI)	SE	*P* value
Out of hours (true/false)	1.41 (0.95-2.11)	0.012	.09
Age	1.07 (1.05-1.09)	0.434	<.001
Chest pain (true)^a^	0.59 (0.39-0.89)	0.012	.008
Activator nurse diagnosis suggesting acute myocardial infarction (true)	1.26 (0.73-2.16)	0.012	.39
Computer diagnosis suggesting acute myocardial infarction (true)	1.30 (0.78-2.17)	0.013	.31
Activator nurse–computer acute myocardial infarction agreement (true)	0.97 (0.47-2.03)	0.011	.95

^a^42 patients with chest pain died after 1 year, whereas 130 patients without chest pain died after 1 year.

**Table 2:** Odds ratios of variables derived from multiple logistic regression where the response variable was mortality after 30 days.

Variable	Odds ratio (95% CI)	SE	*P* value
Out of hours (true/false)	1.39 (0.90-2.20)	0.012	.17
Age	1.06 (1.04-1.08)	0.434	<.001
Chest pain (true)^a^	0.47 (0.29-0.74)	0.012	.001
Activator nurse diagnosis suggesting acute myocardial infarction (true)	1.06 (0.59-1.87)	0.012	.84
Computer diagnosis suggesting acute myocardial infarction (true/false)	0.86 (0.49-1.57)	0.013	.68
Activator nurse–computer acute myocardial infarction agreement (true)	1.46 (0.65-3.31)	0.011	.35

^a^25 patients with chest pain died after 30 days, whereas 92 patients without chest pain died after 30 days.

#### Acute Myocardial Infarction Terminology

Table 3 shows the most frequently used terms by the computer and activator nurses for ECG interpretation to suggest acute myocardial infarction or not suggest acute myocardial infarction. The computer used the term abnormal ECG most frequently, which we classified as not suggesting acute myocardial infarction, whereas activator nurses used the term high take-off for interpreting the ECG, which we classified as not suggesting acute myocardial infarction. Moreover, the computer used the term acute myocardial infarction most frequently for suggesting acute myocardial infarction, and activator nurses used the terms ST depression or ST-elevation for suggesting acute myocardial infarction. Overall, the activator nurses used 45 unique terms to interpret the ECG as not suggestive of acute myocardial infarction and used 19 different terms in suggesting acute myocardial infarction. In contrast, the computer used 59 different terms to interpret the ECG as not suggestive of acute myocardial infarction and 60 unique terms in suggesting acute myocardial infarction.

**Table 3:** Frequently used terms by computer and activator nurses for suggesting or not suggesting acute myocardial infarction.

Classification and rank^a^		Computer		Activator nurse	
		Interpretation term	Patients, n (%)	Interpretation term	Patients, n (%)
**Suggests acute myocardial infarction^b^**					
	1	“acute myocardial infarction”	337 (47)	“Ste”	159 (25)
	2	“inferior infarct”	23 (3)	“St depression”	125 (20)
	3	“anterior injury”	34 (5)	“twi”	129 (20)
**Does not suggest acute-myocardial infarction^c^**					
	1	“abnormal ECG”	382 (51)	“nil acute”	377 (45)
	2	“LBBB”	108 (15)	“high take-off”	187 (23)
	3	“borderline ECG”	41 (5.5)	“RBBB”	59 (7)

^a^Terms with low frequencies (1 or 2) are not included.

^b^n=722 for Computer; n=634 for Activator nurse.

^c^n=742 for Computer; n=830 for Activator nurse.

#### Interpretation Terminology

Activator nurses were more consistent in their nomenclature in suggesting acute myocardial infarction. In contrast to the activator nurse, the computer used a greater range of nomenclature in suggesting acute myocardial infarction (Table 3). The terms with low frequencies (1 or 2 instances) are not included.

## Discussion

### Principal Findings

The level of agreement between human and computer ECG interpretation for acute myocardial infarction regarding patients who were declined for primary percutaneous coronary intervention is an interesting research area for clinicians. It unveils useful insights. In this study, we analyzed an anonymized data set from Altnagelvin Hospital (Northern Ireland, United Kingdom) of patients who were declined for primary percutaneous coronary intervention from January 2015 to December 2017. The total study population consisted of 1464 patients who were declined for a primary percutaneous coronary intervention (996/1464, 68.0% men). The decision was appropriate for all patients; none of the patients who were declined for primary percutaneous coronary intervention experienced an acute ST-elevation myocardial infarction. More declined patients were referred out of hours 66.3% (971/1464). Out of all 1464 declined patients, 117 (8.0%) patients died within 30 days, and a total of 174 (11.8%) patients died within 1 year. Furthermore, the 1-year mortality rate was highest if the patient was referred at 4 AM (7/12, 58.3%). This is not surprising as patients who are less sick are less likely to present in the middle of the night.

Human and computer ECG interpretations did not have a high level of agreement, and the computer tended to suggest acute myocardial infarction more often than the specialist activator nurses, especially for the declined patients. A total of 722/1464 (49.3%) computerized diagnoses suggested acute myocardial infarction, whereas only 634/1464 (43.3%) activator nurse diagnoses suggested acute myocardial infarction (*P*=.001). However, the activator nurse interpreted that ECGs suggested acute myocardial infarction more often during the middle of the night (12 AM to 6 AM: mean 53%, SD 5.3%) than in daytime hours (mean 41%, SD 6.6%; *P*=.001). In contrast, the computer interpretation did not show much difference for hours of the day; for the middle of the night (12 AM to 6 AM), the average acute myocardial infarction ECG interpretation was 47% (SD 4.7%), and for the rest of the hours of the day, the average acute myocardial infarction ECG interpretation was 50% (SD 5.2%). We speculate that there may be human bias at night—the activator nurses tend to overidentify acute myocardial infarction during the night possibly because they are forced to make a decision when there are fewer consultants or clinicians available for a second opinion.

Prior research stated that major problems in computer interpretation were the interpretation of rhythm disturbances and the diagnosis of acute myocardial infarction, T-wave changes, and ventricular hypertrophy [19]. Researchers also found that there was a considerable difference in accuracy between 3 different computer systems [19].

There were only 342/1464 (23.3%) patients for whom there was human and computer agreement that there was an acute myocardial infarction. There was agreement more often for not being acute myocardial infarction (450/1464, 30.7%; *P*<.001). The overall agreement rate was only 54.1% (792/1464). The maximum agreement between activator nurses and the computer occurred from 2 PM to 4 PM (139/231, 60.2%). There were 292/1464 (19.9%) patients for whom the computer did not suggest an acute myocardial infarction but the activator nurse did, and 380/1464 (26.0%) patients for whom the activator nurse identified an acute myocardial infarction but the computer did not. The peak disagreement rate between activator nurse and computer occurred at 11 AM (53/98, 54.1%). The result shows that the computer interpreted ECGs as suggesting acute myocardial infarction more often than activator nurses. Activator nurse–computer agreement was poor (Cohen κ=0.08, *P*=.001). Activator nurses seemed to use fewer terms, whereas the computer used almost 60 different terms suggesting acute myocardial infarction. Previous studies [20] show that there is significant interobserver variability that results in false positives and false negatives. There is a higher rate of discordance among clinically significant ECGs [21].

Additionally, 556 out of 1464 (38.0%) patients who were declined had chest pain. More patients reported chest pain during the middle of the night, between 4 AM and 5 AM (34/55, 61.8%; *P*=.001). This could be because underlying medical conditions and obstructive sleep apnea can be a trigger for myocardial infarction [22]. For logistic regression analysis, both age and chest pain were the only independent variables that were statistically significant in predicting mortality after 30 days (*P*<.001 and *P*=.001, respectively) and 1 year (*P*<.001 and *P*=.008, respectively). Another trending variable was *out of hours*, which increased the odds of 1-year mortality. Being referred out of hours was more predictive for 1-year mortality than 30-day mortality. This could be because not all referral resources were available out of hours. The odds of mortality within 30 days and 1 year of referral were lower in patients with chest pain than in those patients without chest pain. This might be because people with chest pain call for help sooner and receive the appropriate treatment. People without chest pain are more likely to be misdiagnosed.

### Limitations

This was a retrospective analysis. The results are based on a single data set from one hospital in Northern Ireland, which can limit the results; the results may not be generalizable for the overall population and primary percutaneous coronary intervention services.

### Policy and Practical Implications

Algorithms to detect acute myocardial infarction need to be improved. More ECG data are needed for training ECG interpretation algorithms. Perhaps deep learning and neural networks can be used with the ECG interpretation algorithms for more accurate results. In addition, enhanced training and education can provide nurses and activator nurses with support for enhanced ECG interpretation capabilities. ECG interpretation in a primary percutaneous coronary intervention service should be more sophisticated and rely upon more than ST-elevation myocardial infarction criteria. Algorithms could be trained to read ECGs using ECG data sets that have a better ground truth for a fully occluded artery. This label could be based on immediate angiographic findings from ST-elevation myocardial infarction and non–ST-elevation myocardial infarction patients.

### Conclusion

The agreement between computerized and human ECG interpretation was poor for patients who were declined for primary percutaneous coronary intervention. This uncertainty makes it difficult to accept or decline referrals. The results show that the computer suggests acute myocardial infarction more often than activator nurses for declined patients. Work is needed to improve computer and human decision making to ensure that patients are referred to the correct treatment facility for time-critical therapy. In future, there might be comparison among the computer human agreement between male and female patients and various age groups. We believe that this might be an interesting research question.

### Clinical Perspectives 

The 12-lead ECG remains the mainstay in assessing patients with suspected coronary artery occlusion. However, despite improvements in the quality of data acquisition and computer-generated reports, the accuracy of using ECG to diagnose occluded coronary arteries remains suboptimal. There remains a need for improved computer-generated interpretation, which may need to consider patient factors such as sex, age, risk factors, and ongoing symptoms. Including these factors could improve diagnostic accuracy and help triage patients to the best possible treatment. What is unknown is whether this would lead to better clinical outcomes in terms of reduced infarction size and better survival in patients having a myocardial infraction. This study described the interaction and ECG interpretation agreement rate between humans and computers and how they might have an impact on outcomes.

## Abbreviations

CathLab: catheterization laboratory
ECG: electrocardiogram
OR: odds ratio
